# Case Report: Immune-related colitis complicated by *Clostridioides difficile*-associated colitis

**DOI:** 10.3389/fimmu.2026.1914463

**Published:** 2026-10-06

**Authors:** Wenjuan Chen, Wanqian Zhang, Chaoqun Sun, Lin Meng, Xiujuan Qu, Ying Chen

**Affiliations:** 1 Department of Medical Oncology, The First Hospital of China Medical University, Shenyang, China; 2 Key Laboratory of Anticancer Drugs and Biotherapy of Liaoning Province, The First Hospital of China Medical University, Shenyang, China; 3 Liaoning Province Clinical Research Center for Cancer, The First Hospital of China Medical University, Shenyang, China; 4 Key Laboratory of Precision Diagnosis and Treatment of Gastrointestinal Tumors, Ministry of Education, The First Hospital of China Medical University, Shenyang, China

**Keywords:** case report, *Clostridioides difficile*, immune-related adverse events, immune-related colitis, microsatellite instability-high gastric cancer

## Abstract

**Background:**

Immune checkpoint inhibitors (ICIs) have become the standard treatment for patients with advanced microsatellite instability-high (MSI-H) gastric cancer. However, the incidence of immune-related adverse events (irAEs) has also increased accordingly. Immune-related colitis (IRC) is among the most common gastrointestinal manifestations of these irAEs.

**Case presentation:**

This report describes a patient with advanced MSI-H gastric cancer who received immunotherapy and developed Grade-3 IRC during treatment. The patient recovered following corticosteroid therapy. Diarrhea recurred after discontinuation of corticosteroids, a condition that may be misdiagnosed as a recurrence of IRC. Following a multidisciplinary team consultation, the patient was clinically diagnosed with Clostridioides difficile infection (CDI) based on symptoms and stool testing, and showed rapid improvement after vancomycin therapy. The patient achieved a durable complete clinical response lasting more than 5 years without further systemic anticancer therapy.

**Conclusion:**

This case illustrates that immunotherapy may confer long-term benefit in MSI-H advanced gastric cancer. Notably, the occurrence of immune-related adverse events coincided with sustained tumor control, suggesting a potential association that merits further exploration. However, when diarrhea recurs after discontinuation of corticosteroids, clinicians must remain highly vigilant regarding the overlap and differentiation between the reactivation of IRC and infectious colitis, the involvement of a multidisciplinary team is particularly crucial.

## 1 Introduction

Gastric cancer (GC) is one of the most prevalent malignant tumors worldwide. Its incidence and mortality rates consistently rank among the highest globally (1). Mismatch repair deficiency/microsatellite instability-high GC accounts for only approximately 6% of advanced GC cases (2). Due to its unique immune microenvironment and high tumor mutational burden, this subgroup exhibits a particularly strong response to immunotherapy. Multiple clinical studies confirmed that this patient subgroup achieves an objective response rate (ORR) of 50%–70% after immunotherapy, with a median progression-free survival (PFS) of 11–18 months and a median overall survival (OS) exceeding 30 months (3–5). However, the incidence of immune-related adverse events (irAEs) has also increased significantly, with Immune-related colitis (IRC) being one of the most common irAEs in the gastrointestinal system (6). According to the latest clinical data, the incidences of diarrhea and colitis induced by anti-programmed death-1 (PD-1)/programmed death-ligand 1 (PD-L1) antibodies are 10% and 2%, respectively, whereas the incidences induced by the CTLA-4 inhibitor ipilimumab are 33% and 7%, respectively, with low rates of Grade 3–4 diarrhea/colitis (7). The clinical manifestations of IRC are diverse, ranging from mild diarrhea to severe abdominal pain, hematochezia, and fever, which can be life-threatening in severe cases (8).

Grade-1 immune-related colitis (IRC) is primarily managed with symptomatic support, Grade-2 IRC is initially treated with corticosteroids, while Grade 3–4 IRC warrants immediate and permanent discontinuation of ICIs. Notably, patients who received immunotherapy often present with different degrees of immunocompromised status. Coupled with the use of corticosteroids or immunosuppressants to manage irAEs, their risk of infection is further increased. Therefore, when diarrhea reoccurs after the remission of IRC, in addition to considering the flare of IRC, the possibility of infectious colitis must be underscored. Common pathogens include cytomegalovirus, Epstein-Barr virus, bacteria, and parasites, among which *Clostridioides difficile* infection (CDI) is of particular concern. In a large retrospective study, PCR testing for *Clostridioides difficile* was conducted on 605 patients who developed diarrhea following ICI treatment, the results showed that 111 cases (18.3%) tested positive (9). The clinical symptoms of CDI overlap with those of IRC to a significant extent, and both can occur following ICI therapy, making their differentiation extremely challenging. Once misdiagnosed, inappropriate use of corticosteroid may exacerbate the infection, while blind administration of antibiotics can miss the opportunity to control immune inflammation.

Although there have been case reports of PD-1 inhibitor-induced IRC, the occurrence of CDI following the remission of IRC is relatively rare. This case report refers to a patient with MSI-H advanced GC who developed Grade-3 IRC after anti-PD-1 immunotherapy. After alleviation with corticosteroid therapy, the patient experienced severe diarrhea again. Through a systematic review of medical history, pathogenic testing, and differential diagnosis, the patient was clinically diagnosed with CDI and achieved a favorable outcome following standardized anti-infective treatment with vancomycin.

## 2 Case presentation

The clinical course is summarized in **Figure 1**. A 60-year-old woman presented with “pain in the left lower back and buttock.” An MRI of the hip showed multiple areas of T2 hyperintensity in the left acetabulum and ischium, and malignancy could not be excluded. A left ischial needle biopsy was performed, and pathological diagnosis coincided with metastatic adenocarcinoma. Positron emission tomography-computed tomography revealed: 1. Unevenly increased density and metabolism in the left acetabulum and ischium, highly suggestive of malignant metastatic lesions; 2. Wall thickening with elevated metabolism in the gastric cardia, highly suggestive of malignancy, accompanied by multiple small perilesional lymph nodes without increased metabolism. Gastroscopy demonstrated an ulcerative lesion on the posterior wall of the cardia, involving one-third of the circumference, measuring 30 mm × 30 mm, with an uneven base, white coating, and surrounding annular elevated mucosa (**Figure 2**). Pathological diagnosis: cardiac adenocarcinoma. IHC: EBV(-), CPS < 1 (22C3), HER2: 0, MLH1 (loss), PMS2 (loss), MSH6 (+), MSH2 (+). Multi-gene testing: MSI-H, high tumor mutational burden (TMB, 65.28 Muts/Mb).

**Figure 1:**
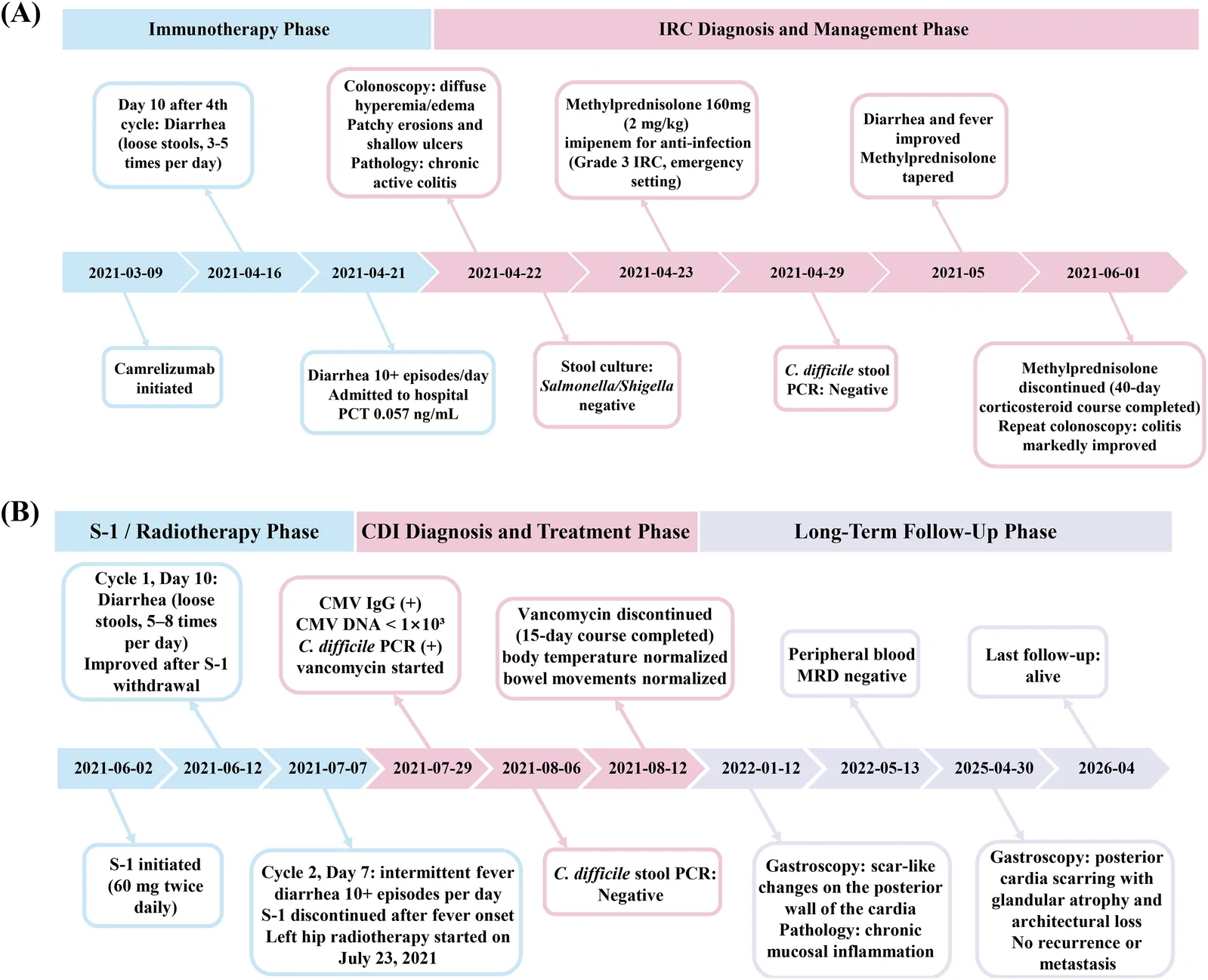
Clinical timeline of the patient’s disease course. The timeline is divided into two panels. **(A)** Anti- PD- 1 therapy and diagnosis/management of immune-related colitis (IRC). **(B)** S- 1 chemotherapy, radiotherapy, recurrent diarrhea, and CDI diagnosis/treatment. IRC, immune-related colitis; PCT, procalcitonin; *C. difficile*, *Clostridioides difficile*; CDI, *Clostridioides difficile* infection; CMV, cytomegalovirus; MRD, minimal residual disease.

**Figure 2:**
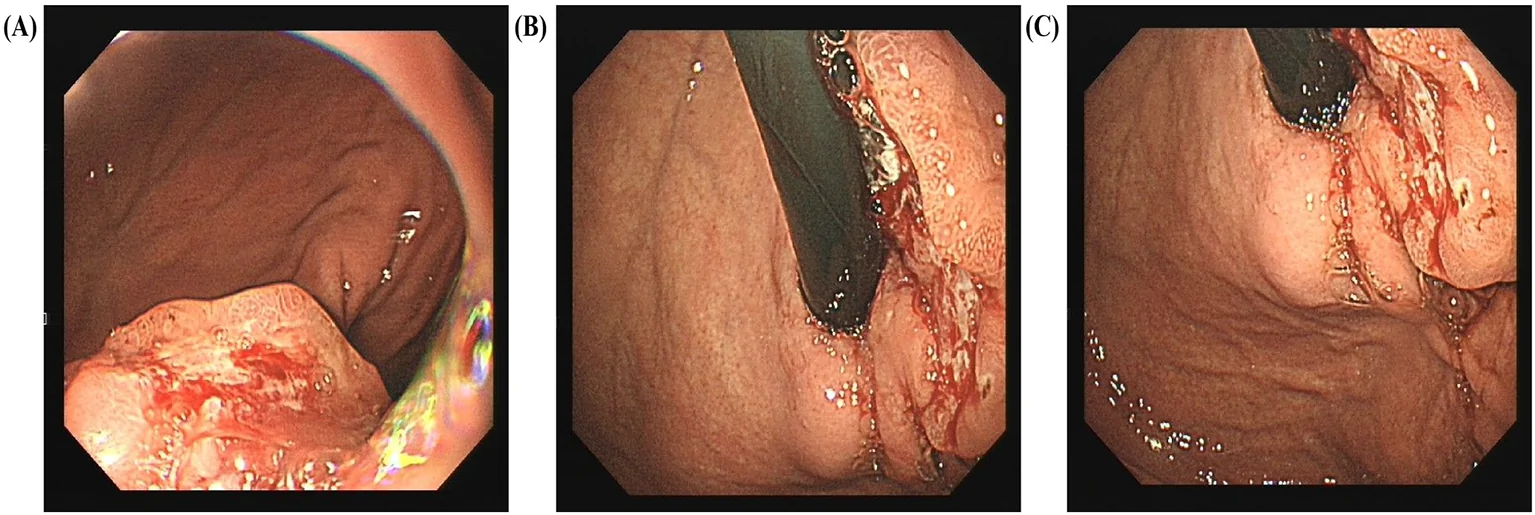
**(A–C)**: Cardia: an ulcerative lesion is seen on the posterior wall, involving about one-third of the circumference, measuring approximately 3.0 × 3.0 cm, with an uneven base and white coating. The surrounding mucosa shows a mound-like elevation. The proximal aspect of the lesion involves the Z-line, and the distal margin is approximately 45 cm from the incisors.

On March 9, 2021, the patient received camrelizumab 200 mg with a biweekly schedule. After three cycles, the efficacy evaluation was one involving stable disease. Ten days after the fourth cycle, the patient developed unprovoked pasty stools, over 10 times per day, accompanied by abdominal pain and intermittent fever (maximum body temperature 37.8 °C). She was admitted on April 21, 2021. Blood routine results: WBC 8.64 × 10^9^/L; neutrophils 5.8 × 10^9^/L; neutrophil percentage 67.1%; PCT 0.057 ng/mL; CRP 60 mg/L. Stool routine: occult blood positive, WBC (+++), red blood cell (RBC) (++++), bacillus/coccus ratio 1:10. Colonoscopy on 22 April 2021 showed diffuse congestion and oedema in the intestinal tract, with patchy erosions and superficial ulcers, and a polyp 150 mm from the anal verge. Colonoscopy pathology: (transverse) colon chronic active inflammation (moderate) with erosion, and mild reactive atypia in a few glands (**Figure 3**). Gastroscopy showed the cardiac lesion was reduced to quarter-circumferential involvement, with a flat base, thin white coating, and regular surrounding mucosa, evincing the improvement compared to previous findings. On April 22, 2021, stool testing was negative for *Salmonella* and *Shigella*. Given the suspected IRC, methylprednisolone 160 mg (2 mg/kg) was initiated on April 23, 2021, along with imipenem for 3 days of anti-infective therapy, somatostatin, and fluid resuscitation. The patient’s diarrhea and fever gradually improved, and imipenem was discontinued thereafter. On April 29, 2021, stool testing for *C. difficile* was negative. The corticosteroid regimen was described as follows: intravenous methylprednisolone 160 mg qd for five days, then gradually tapered to 40 mg qd for three days, switched to oral methylprednisolone 32 mg qd, and tapered by 8 mg every 3–5 days. Methylprednisolone was discontinued on June 1, 2021, with a total duration of 40 days.

**Figure 3:**
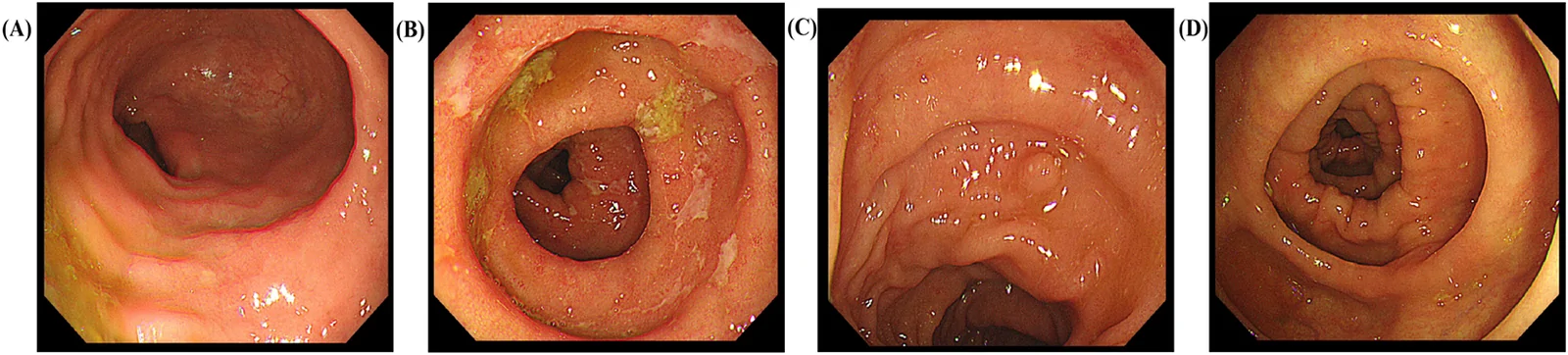
Ascending colon **(A)**: the mucosa shows scattered patchy hyperemia and erosions. Transverse colon **(B)**: The mucosa shows localized edema, with patchy hyperemia and erosions. Sigmoid colon **(C)**: The mucosa shows scattered patchy hyperemia and erosions; a sessile polyp, approximately 0.5 cm in size and smooth, is seen about 15 cm from the anal verge. Rectum **(D)**: The mucosa shows scattered patchy hyperemia and mild edema.

The patient was readmitted on June 1, 2021. A follow-up colonoscopy showed spot-like congestion and erosion in the ileocecal region, cecum, transverse colon, descending colon, sigmoid colon, and rectum, evincing significant improvement compared to previous findings. The patient has MSI-H (microsatellite instability-high) gastric cancer. Although the benefit of S-1 (tegafur, gimeracil, and oteracil potassium; an oral fluoropyrimidine) monotherapy is expected to be limited, given safety considerations, S-1 was started on June 2, 2021, at a dose of 60 mg twice daily, with a 2-weeks-on, 1-week-off schedule as one cycle. Concurrently, radiotherapy was delivered to the left hip bone metastasis from July 23, 2021, to September 2, 2021, with a dose of 2 Gy per fraction to the gross tumor volume (GTV) for a total of 30 fractions. On day 10 of the first S-1 cycle, the patient developed diarrhea (5–8 times/day, pasty stools), which alleviated after drug withdrawal. On Day 7 of the second cycle, the patient developed intermittent fever, after which S-1 was discontinued. The stool frequency subsequently increased to more than 10 episodes per day, with symptoms similar to before. The patient was subsequently admitted to the hospital.

Upon admission, blood routine, stool routine, CRP, and PCT were essentially normal; the stool bacillus/coccus ratio was 1:5, cytomegalovirus (CMV) IgG antibody was positive with DNA quantification < 1 × 10^3^, and stool PCR for toxigenic *Clostridioides difficile* DNA was positive for toxin B gene. The patient’s history of corticosteroid therapy was a significant risk factor for bacterial infection, and the last immunotherapy administration was 3 months prior. Based on the clinical presentation, the patient’s diarrhea was attributed to CDI. On July 29, 2021, administration of oral vancomycin (125 mg every six hours) was initiated. Defecation was significantly improved, and body temperature normalized. On August 6, 2021, *Clostridioides difficile* testing returned negative results. After a total of 15 days of vancomycin therapy, the patient recovered and was discharged.

The patient did not receive further anti-tumor therapy thereafter. On January 12, 2022, gastroscopy showed changes in scarring on the posterior wall of the cardia (**Figure 4**), with pathology indicating chronic mucosal inflammation. Subsequent follow-up gastroscopies consistently showed scar changes. On May 13, 2022, peripheral blood minimal residual disease testing was negative. The patient is currently alive, receiving imaging examinations every six months with gastroscopy annually. On April 30, 2025, gastroscopy demonstrated scar-like changes on the posterior wall of the cardia, with atrophy and disappearance of glandular ductal structures. There was no recurrence or metastasis observed. The last follow-up was in April 2026.

**Figure 4:**
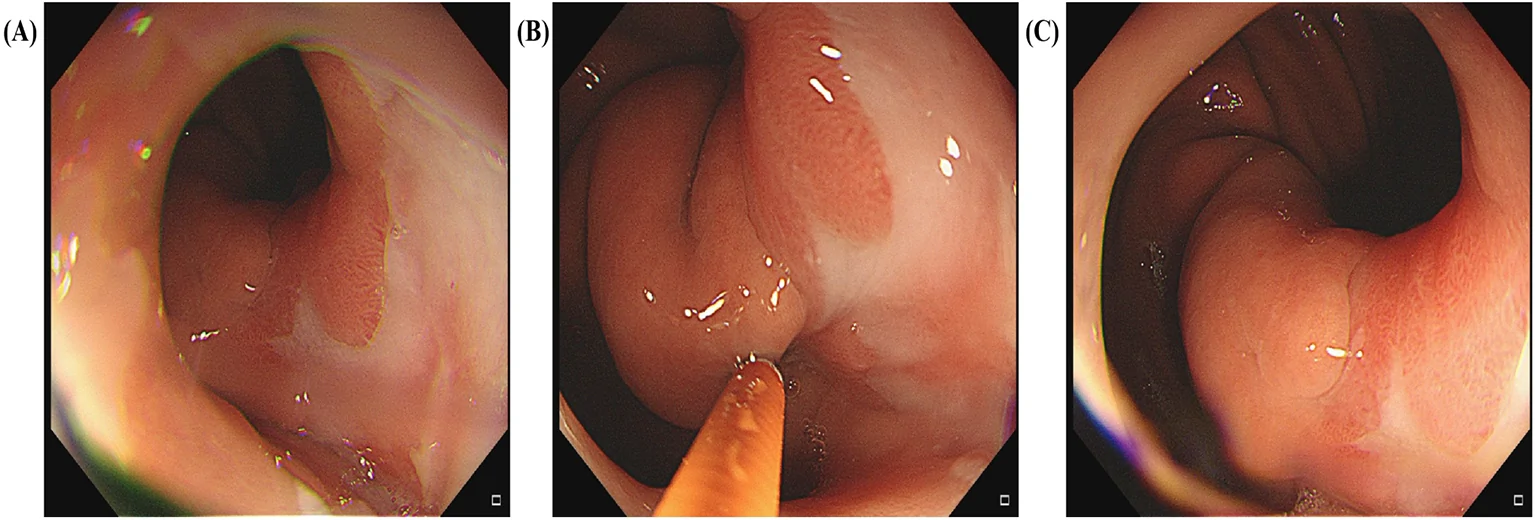
**(A–C)**: Esophagus-cardia: a scar is seen on the posterior wall of the cardia, measuring approximately 1.5 × 1.5 cm, with no ulcer or erosion. Under narrowband imaging (NBI), the surface structure appears relatively regular, and the consistency is slightly firm. The Z-line of the esophagus is approximately 44 cm from the incisors, and the upper margin of the scar is approximately 44 cm from the incisors.

## 3 Discussion

In recent years, the application of ICIs in tumour immunotherapy has achieved breakthrough progress. As ICI application becomes increasingly widespread, reports of their irAEs have gradually increased (10). Camrelizumab is a humanized anti-PD-1 monoclonal antibody that binds to the PD-1 receptor on T cells, blocking its interaction with ligands PD-L1 and PD-L2, thereby disrupting PD-1 pathway-mediated immunosuppression and restoring antitumor immune responses (11). Although the overall incidence of ICI-induced adverse events is relatively high, some adverse events, such as IRC and subsequent complications with CDI, are rare in clinical practice. However, insufficient clinical awareness may lead to severe or even fatal outcomes.

Studies indicate that the gastrointestinal tract is a frequently affected site by irAEs, with diarrhea and colitis being the most typical manifestations. Other manifestations include abdominal pain, hematochezia, nausea, and vomiting. Among ICI-related adverse reactions, the incidence of diarrhea/colitis is 9.47%, and the incidence of Grade-3 (or higher) diarrhea/colitis is 0.59% (12). Accordingly, when this patient developed diarrhea, fever, and abdominal pain following immune checkpoint inhibitor therapy, and conventional fluid resuscitation and antidiarrheal treatment proved ineffective, colonoscopy was performed. Endoscopic examination revealed diffuse mucosal hyperemia and edema, with patchy erosions and shallow ulcers, predominantly in the rectum; histopathological examination showed moderate chronic active colitis with erosions and mild glandular atypia, and no viral inclusion bodies, fungal elements, or parasitic organisms were identified—findings consistent with immune-related colitis. In addition, the PCT level was low (0.057 ng/mL), which did not support bacterial infection, and stool PCR for *Clostridioides difficile*, collected on the day of admission, was also negative. Based on the integration of clinical presentation, endoscopic and histopathological evidence, and the exclusion of infectious and other etiologies, the patient was diagnosed with IRC. According to the Common Terminology Criteria for Adverse Events Version 5.0, the patient was evaluated as having grade 3 PD-1 inhibitor-induced IRC, with corticosteroids as the first-line treatment (13). Following specialist evaluation, the patient was initiated on corticosteroid therapy at an initial dose of 2 mg/kg/day and gradually tapered, leading to significant symptom relief.

Under the circumstance where first-line treatment was effective and IRC had been in remission for two months, the patient subsequently developed recurrent diarrhea. Should this only be considered a flare-up of IRC? Patients with cancer who develop irAEs are often treated with corticosteroids or even immunosuppressants, placing the body in an immunosuppressed state and making them highly susceptible to concurrent infections. Especially among cancer patients receiving immunotherapy, the risk of *Clostridioides difficile*-associated diarrhea is significantly elevated, accounting for 18.3% of those treated with ICIs (9). Therefore, we added testing for *Clostridioides difficile* to investigate the etiology of the patient’s diarrhea, and the result was confirmed. Subsequently, based on US and European guidelines for patients with *Clostridioides difficile*-associated diarrhea, the patient’s condition was improved significantly after vancomycin treatment (14, 15). If this had been missed and simply ascribed to IRC, re-initiating immunosuppressive therapy would likely have further exacerbated the diarrhea and delayed appropriate treatment.

The differential diagnosis of recurrent diarrhea required differentiation among S-1-induced diarrhea, IRC relapse, and CDI. Regarding S-1: a meta-analysis of four randomized controlled trials demonstrated that the incidence of grade ≥ 3 diarrhea with S-1 monotherapy was only approximately 4% (16), indicating that severe diarrhea is not a common toxicity of S-1. In our patient, S-1 had been discontinued before admission, yet diarrhea persisted and worsened, and responded rapidly to vancomycin—further arguing against S-1 as the primary cause. Regarding IRC relapse: although the immunomodulatory effects of PD-1 inhibitors may persist for months after cessation, and thus IRC relapse remains theoretically possible, this diagnosis was not supported by the clinical course—the patient achieved complete remission with vancomycin alone without corticosteroid re-exposure, which is inconsistent with the typical clinical presentation of IRC relapse (flares generally require corticosteroid re-intervention for control). Regarding CDI: robust direct microbiological evidence supported this diagnosis—positive stool PCR for toxigenic *C. difficile* DNA (meeting IDSA criteria), rapid improvement following vancomycin, and negative conversion on repeat PCR, with a highly specific therapeutic response. Notably, the patient had multiple risk factors for CDI: a 40-day history of corticosteroid therapy—a well-established risk factor for bacterial infections (17); short-course imipenem use during the initial hospitalization, also a potential risk factor for CDI, with literature reporting a 5.31-fold increased risk associated with carbapenem treatment (18); and a 3-month interval since the last immunotherapy—although this leaves open the theoretical possibility of IRC relapse, it also implies that the patient’s intestinal immune homeostasis had not fully recovered. These factors, in combination, may have collectively created a permissive gut environment for CDI to develop following S-1-related mucosal injury. In conclusion, although no tissue samples were available for histological evaluation, the integration of clinical presentation, direct microbiological evidence, specific therapeutic response, and host risk factors established CDI as the predominant etiology of the recurrent diarrheal episode, rather than IRC relapse.

This patient who had MSI-H GC experienced a complicated course during ICI monotherapy. She developed not only IRC but also subsequent *Clostridioides difficile*-associated colitis in an immunocompromised state. However, this did not affect the efficacy of subsequent treatment. The patient achieved imaging complete response (CR), endoscopic and biopsy-confirmed complete remission, and a negative peripheral blood minimal residual disease test. Over a follow-up period of four years and eight months, the most recent endoscopic and biopsy findings remained consistent with a complete clinical response. The occurrence of immune-related adverse events (irAEs) in this patient was temporally associated with sustained tumor response. While irAEs have been linked to favorable outcomes in some ICI-treated cohorts (19), causality cannot be established from a single case, and the predictive value of irAEs for treatment response remains to be validated in prospective studies.

## 4 Limitations

Several limitations of this case should be acknowledged. First, although the second diarrheal episode responded rapidly to vancomycin and was clinically attributed to CDI, the diagnosis was based primarily on predisposing risk factors, clinical presentation, and positive stool PCR for toxigenic *C. difficile* DNA. PCR alone cannot distinguish active infection from asymptomatic colonization, further confirmatory testing—such as toxin protein assays, stool culture, or endoscopic/histopathological evaluation—would help to avoid misdiagnosis. Second, the prognostic value may differ across organ-specific irAEs: studies have shown that cutaneous and endocrine irAEs are associated with survival benefits (20), whereas the prognostic significance of gastrointestinal irAEs, such as colitis, remains uncertain. In our patient, grade 3 IRC required corticosteroid intervention and was followed by CDI, which further complicates the attribution of long-term survival benefit. Therefore, the temporal association observed in this single case between irAEs and durable tumor response does not imply causation, and any conclusions should be regarded as hypothesis-generating rather than confirmatory.

## Data Availability

The original contributions presented in the study are included in the article/supplementary material. Further inquiries can be directed to the corresponding authors.
